# Self-injury and externalizing pathology: a systematic literature review

**DOI:** 10.1186/s12888-017-1326-y

**Published:** 2017-05-03

**Authors:** Gergely Meszaros, Lili Olga Horvath, Judit Balazs

**Affiliations:** 1 Semmelweis University, Mental Health Sciences School Of Ph.D., Üllői út 26, Budapest, 1085 Hungary; 2 Vadaskert Child Psychiatry Hospital and Outpatient Clinic, Lipótmezei út 1-5, Budapest, 1021 Hungary; 3 Doctoral School of Psychology, Eötvös Loránd University, Izabella utca 46, Budapest, Hungary; 4 Institute of Psychology, Eötvös Loránd University, Izabella utca 46, Budapest, Hungary

**Keywords:** NSSI: nonsuicidal self-injury, DSH: deliberate self-harm, SIB: self-injurious behaviour, self-injury, externalization, ADHD: attention deficit hyperactivity disorder, CD: conduct disorder, ODD: oppositional defiant disorder, psychopathology, psychiatric disorder

## Abstract

**Background:**

During the last decade there is a growing scientific interest in nonsuicidal self-injury (NSSI). The aim of the current paper was to review systematically the literature with a special focus on the associations between self-injurious behaviours and externalizing psychopathology. An additional aim was to review terminology and measurements of self-injurious behaviour and the connection between self-injurious behaviours and suicide in the included publications.

**Methods:**

A systematic literature search was conducted on 31st December 2016 in five databases (PubMed, OVID Medline, OVID PsycINFO, Scopus, Web of Science) with two categories of search terms (1. nonsuicidal self-injury, non-suicidal self-injury, NSSI, self-injurious behaviour, SIB, deliberate self-harm, DSH, self-injury; 2. externalizing disorder, attention deficit hyperactivity disorder, ADHD, conduct disorder, CD, oppositional defiant disorder, OD, ODD).

**Results:**

Finally 35 papers were included. Eleven different terms were found for describing self-injurious behaviours and 20 methods for measuring it. NSSI has the clearest definition. All the examined externalizing psychopathologies had strong associations with self-injurious behaviours according to: higher prevalence rates in externalizing groups than in control groups, higher externalizing scores on the externalizing scales of questionnaires, higher symptom severity in self-injurious groups. Eight studies investigated the relationship between suicide and self-injurious behaviours and found high overlap between the two phenomena and similar risk factors.

**Conclusions:**

Based on the current findings the association between externalizing psychopathology and self-injurious behaviours has been proven by the scientific literature. Similarly to other reviews on self-injurious behaviours the confusion in terminology and methodology was noticed. NSSI is suggested for use as a distinct term. Further studies should investigate the role of comorbid conditions in NSSI, especially when internalizing and externalizing pathologies are both presented.

## Background

In the past decade self-injury became a hot topic in scientific literature. Nonsuicidal self-injury (NSSI) became an individual diagnosis in the 5th edition of the Diagnostic and Statistical Manual of Mental Disorders [1]; for the present it can be found only in Section III in the chapter ‘Conditions for Further Study’. The definition for NSSI in DSM-5 follows the main instructions of the definition of the ‘International Society for the Study of Self-injury’ (ISSS), which was made in 2007 [2]. This definition emphasizes the deliberate, nonsuicidal purpose of the self-injurious act, which is not socially sanctioned. It underlines the importance to distinguish it from drug overdoses, culturally sanctioned behaviours (e.g. piercings), and repetitive, stereotypical forms among people with developmental disorders [2]. The DSM-5 suggests as a criterion of NSSI, that self-injurious acts should happen on at least 5 days in the past year. Moreover the DSM-5 underlines the non adaptive ‘coping strategy’ nature of NSSI: the individual who engages in NSSI must have the aim to get to a better emotional state after the action [1].

In the 4th edition of DSM [3], deliberated NSSI can only be found as a symptom of borderline personality disorder (BPD), however clinicians can meet a lot of patients with self-injury without BPD as well. Glenn and Klonsky [4] found in an adolescent psychiatric sample that the coexistence of NSSI and BPD is not more common than the coexistence of any other Axis I diagnoses and NSSI. Zetterqvist [5] found that in the population who met NSSI DSM-5 criteria there are more general psychopathology and impairment, than in the population who did not present NSSI behaviour or in the population who present NSSI behaviour, but did not meet the DSM-5 criteria.

Several risk factors of NSSI are described, such as prior history of NSSI, cluster b personality, hopelessness, female gender, depression, prior suicidal thought/behaviour, exposure to peer NSSI, eating disorder, abuse, etc. [6].

Many studies examined the association between NSSI and internalizing pathology; recently Bentley et al. [7] summarized these studies in their meta-analytic review: all the examined emotional disorders had an increased odds ratio for NSSI, except for bipolar disorder and social anxiety disorder. The association was the strongest with panic and post-traumatic stress disorder, otherwise there were no significant differences between the emotional disorders. Inconsistent methodologies are emphasized, such as the wide range of instruments used to assess NSSI and the multiple terms for self-injury, including NSSI.

The association between externalizing pathology and NSSI are understudied. Although Fox et al. [6] found that the odds ratios of ‘misc externalizing symptoms’ is higher (1.68) than ‘misc internalizing symptoms’ (1.37). According to our knowledge, there is only one review that investigated a specific externalizing disorder (attention deficit hyperactivity disorder – ADHD) and its association with self-injury, and found that ADHD is a risk factor of self-harm [8].

Therefore, our aim is to systematically review the studies that examined the association between self-injurious behaviour and externalizing psychopathology. Not only ‘NSSI’ was chosen as a search word for self-injurious behaviour, because the scientific literature is very confused about the terms [9]. Moreover, e.g. Brunner, Kaess et al. [10] noted this important aspect, often in clinical practice it is hard to distinguish the purpose of a self-injurious act.

Before investigating the main aim of our study – 1) prevalence rates, odds ratios or other associations between externalizing pathology and self-injury – it seemed to be important to review: 2) terminology and definitions of self-injurious behaviour, and 3) methods for measuring it. Finally, we examined: 4) associations between self-injury and suicide among the included publications.

## Methods

**Fig. 1:**
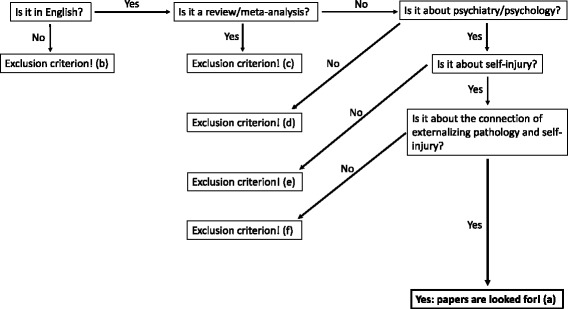
*QUORUM flowchart*

A systematic literature search was made on 31th December 2016 in five computerised literature databases: PubMed, OVID Medline, OVID PsycINFO, Scopus, Web of Science. Search terms were: ‘(nonsuicidal self-injury OR non-suicidal self-injury OR NSSI OR self-injurious behaviour OR SIB OR deliberate self-harm OR DSH or self-injury) AND (externalizing disorder OR attention deficit hyperactivity disorder OR ADHD OR conduct disorder OR CD OR oppositional defiant disorder OR OD OR ODD)’. We found 902 papers in PubMed, 127 papers in OVID Medline, 120 papers in OVID PsycINFO, 116 papers in Web of Science and 16 papers in Scopus, making a total of 1281 studies including duplicates. We used EndNote X7 software for the systematization of the papers. After a duplicate search (both automatic by the software and manual by reading the authors and the titles again) we excluded 222 articles, so altogether we had 1059 individual papers (see the details in Fig. 1).

**Fig. 2:**
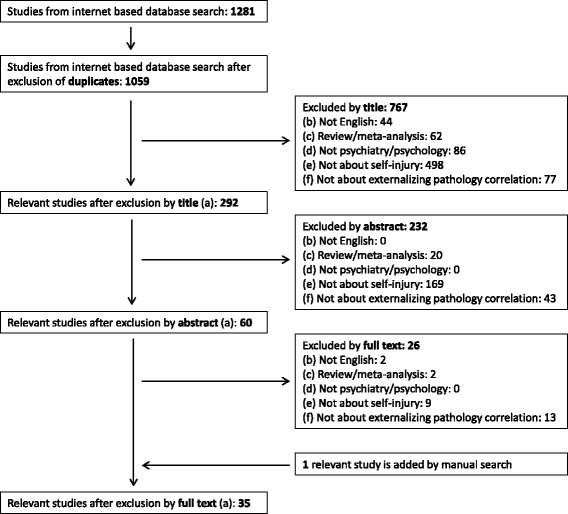
Flowchart of inclusion/exclusion process

The flowchart of the inclusion/exclusion process can be seen in Fig. 2. Inclusion criteria were that studies investigating self-injury and externalizing pathology (a), are written in English (b), and reported original data (c). Exclusion criteria were studies: that were not written in English (b), were review/meta-analysis (c), were not about psychiatry/psychology (d), were not about self-injury (e), were not about externalizing pathology correlation (f).

A QUORUM flowchart of the selection process is summarized in Fig. 1. Publications included in the systematic review were selected in three steps: 1) from the 1059 individual papers, based on their titles 767 publications were excluded, 2) from the remaining 292, based on their abstracts 232 more were excluded 3) from the remaining 60 papers, 26 more were excluded after reading their full-texts. Finally, we added 1 paper by manual search. Therefore, a total of 35 papers are included in our systematic review (Table 1). First, each paper’s title, abstract and full-text was investigated by the first author, which process was blinded reviewed by the second author. When there was a disagreement between the first and second authors whether a paper should be included or excluded to the systematic review, a common discussion and a final collective decision was made by all the three authors.

**Table 1:** Included relevant articles examining associations between self-injury and externalizing pathology

Author, year	Country	Design	Age group	No of participants	Terminology for self injurious behaviour	Distinction betwen suicidal or non-suicidal intent	Measurement of self-injurious behaviours	Main results	Externalization pathology and self-injury association
Aglan et al. (2008) [11]	UK	prospective; baseline: adolescents took part in a family intervention; follow up: six years later; self-poisoners	BL: M = 14.5; SD = 1.1; FU: M = 20.8; SD = 1.1	BL: 158; FU: 126	DSP	No	part of the interviewing process, question for DSH	The main risk factor for repeated DSH is MDD. However, adulthood adversity (DSH is included) is influenced by other risk factors such as: child sexual abuse and adolescent hopelessness and conduct disorder.	Conduct disorder is a risk factor in their model to DSH repetition in adulthood. Correlation coefficient: 0.17.
Bacskai et al. (2012) [18]	Hungary	cross sectional, drug dependent patients from outpatient drug centers with and without ADHD	M = 27; SD = 6.3	210	self-injury	Yes	part of the interviewing process, question for self-injury	The highest aggressive tendencies were shown by the ADHD group. ADHD patients had higher levels of depression, suicidality and self-injury.	Physical self-injury is significantly more prevalent among substance dependent patients with ADHD than without ADHD (X^2^ = 25.2; *p* < 0.0001).
Carli et al. (2010) [19]	Italy	cross sectional, incarcerated volunteers	N.A.	1265	self-mutilation	Yes	part of the interviewing process, question for self-mutilation	Psychoticism, extraversion, aggression, hostility and resilience capacity were more frequent among high impulsive subjects. They engaged in self-mutilation more frequently, substance use disorder was also more frequent in this group. There was no difference between groups in suicidality.	Prevalence of self-mutilation is significantly higher in high-impulsive group than in low-impulsive group (X^2^ = 9.27; *p* = 0.001), but there is no difference in the level of suicidality.
Cerutti et al. (2011) [20]	Italy	cross-sectional, normal population	13–22; M = 16.47; SD = 1.7	234	DSH	Yes	questionnaire for DSH; DSHI	They found a correlation between all the psychopathologies examined: CD, ODD, BPD, dissociative symptoms. They also found a correlation between DSH and stressful life events (psychological and sexual abuse, natural disasters and serious accidents, the loss of someone important, and the witnessing of family violence or a serious accident).	There was a strong correlation between DSH and CD/ODD (correlation coeffitients: ODD: 0,39; CD: 0.41; *p* < 0.01). There were no differences in the levels of correlation between DSH and CD and DSH and ODD.
Chou et al. (2014) [12]	Taiwan	retrospective, Taiwanian National Healthcare Insurance Database; patients with ADHD, matched controls; 3 years interval	<18; ADHD: M = 8.5; SD = 3.0; HC: M = 8.4; SD = 3.0	3685; 36,850	DSP	No	based on clinical data	50% of the NSSI group engaged in this behaviour once or twice; 36% four times or more. No self-harm group: lowest levels of risk factors, highest levels of protective factors. Adolescents in the NSSI group had fewer depressive symptoms, lower suicidal ideation, greater self-esteem and parental support than the suicide attempt group.	DSP incidence was significantly higher in the ADHD group than in comparsion group. Adjusted hazard ratio: 4.65 (95% CI: 2.41–8.94); *p* < 0.05.
Crowell et al. (2005) [13]	USA	cross-sectional, parasuicidal adolescents and age matched controls	14–18; M = 15.3; SD = 1.1	46 (23 + 23)	parasuicide	No	interview for parasuicide; LPC	Adolescents with a history of parasuicide showed reduced RSA at baseline, greater RSA reactivity during negative mood induction. There were no differences between EDR and PEP. These results support theories that emphasize the importance of emotional dysregulation and impulsivity in parasuicidal teenage girls.	There were significantly more externalization symptoms in the parasuicidal than in the control group. According to parent’s report: F = 39.3; according to self report: F = 26.6; according to teacher’s report: F = 17.2; *p* < 0,001.
Crowell et al. (2012) [21]	USA	cross-sectional with 2 phases: visit1: self-reported questionnaires; visit2: interview, physiological measurement; 3 groups: self-injuring, depressed, control females	13–17; SII group: M = 16.3; SD = 1.0; depressed group: M = 15.4; SD = 1.4; control: M = 16.1; SD = 1.3	75 (25 + 25 + 25)	self-inflicted injury (SII)	Yes	interview for SII; L-SASI same as LPC	The SII group had higher scores on both externalizing psychopathology and emotion dysregulation, and had more attenuated EDR than depressed adolescents. Self-injuring adolescents also had higher scores on borderline pathology. These findings show that there are differences between self-injuring and depressed adolescent girls in aetiology and developmental course.	There were significantly more externalization symptoms in the self-injury group than in the control group or in the depressed group. According to parent’s report: F = 23.7; according to self report: F = 34.5; *p* < 0.001.
Darke and Torok (2013) [22]	Australia	cross-sectional, IDU patients	21–62; M = 37.1; SD = 7.9	300	non-suicidal self-harm	Yes	part of the interviewing process, questions for non-suicidal self-harm	Childhood physical abuse was reported in 74.3%. Independent correlates of non-suicidal self-harm were: female gender, avoided home due to conflict, more extensive polydrug use. Independent correlates of attempted suicide were: severe childhood physical abuse, frequent abuse, avoided home due to conflict, female gender, a positive screen for CD, more extensive polydrug use.	There were no significantly more CD symptoms among drug abuser patients with non-suicidal self-harm than without non-suicidal self-harm. Odds ratio: 1.74 (95% CI: 0.97–3.14); non-significant.
Evren et al. (2014) [23]	Turkey	cross-sectional, normal population	M = 16.7; SD = 6.4	4938	self-harm	Yes	part of a longer questionnaire, questions for self-harm	ADHD symptom scores were higher in females and among adolescents who used tobacco, alcohol and drugs, and had self-harming behaviour and suicidal thoughts in their lifetime. ADHD symptom score was correlated with depression, anxiety, anger and sensation seeking scores, in ANCOVA ADHD symptom score correlated with depression, anxiety, anger, sensation seeking. Alcohol use and suicidal thoughts predicted the severity of ADHD.	Sscores on the questionnaire measuring ADHD symptoms were significantly higher in the self-harming population than in the non-self-harming population (*t* = −15.38; *p* < 0.001).
Feingold et al. (2014) [24]	Israel	cross-sectional, general clinical sample, inpatients	M = 15.8; SD = 4.3	238	self-injury	Yes	based on clinical data	Alcohol-abuse patients had suicide attempts and self-injurious acts more frequently than non-alcohol-abuse patients. Attention-deficit disruptive behaviour disorders, criminal activity and drug use were more common in the abusers group. Median length of stay in hospital was longer in the non-abuser group.	There was significant difference in the prevalence of self-injurious behaviour among alcohol abusers and non-abusers (X^2^ = 7.61; *p* < 0.05). Externalizing pathology was also more common in the alcohol abuser group (X^2^ = 6.29; *p* < 0.05).
Fulwiler et al. (1997) [25]	USA	cross sectional, incarcerated population	Self-mutilators: M = 30; SD = 7.2; suicide attempters: M = 34; SD = 7.3	31	self-mutilation	Yes	based on clinical data	Suicide attempters frequently had adult affective disorder. Self-mutilators more often had a history of childhood hyperactivity and a mixed dysthymia and anxiety that began in childhood or early adolescence.	Childhood ADHD is associated with self-mutilation among adult prisoners (X^2^ = 15.5; *p* = 0.00008).
Garcia-Nieto et al. (2014) [26]	Spain	cross-sectional, clinical sample, inpatients, who had self-injurious behaviours	M = 43.3; SD = 10.3	150	suicide attempt, suicide gesture (NSSI + suicide attempt to communicate with others)	Yes	interview for suicide gestures; SITIBI	Those who engaged in suicidal gestures are more likely to have a personality disorder, especially from Cluster B (histrionic and antisocial). Narcissistic personality disorder was a risk factor for suicide attempt, and borderline personality disorder was a risk factor for both. High impulsiveness was associated with suicide. Suicide attempts and suicide gestures are two distinct phenomena.	Antisocial personality disorder is a risk factor for suicidal gestures. Odds ratio: 2.28 (CI 95%: 1.12–4.64); *p* < 0,05.
Gatta et al. (2016) [27]	Italy	case-control study, neuropsychiatric inpatients and controls	12–17; NSSI group: M = 15.0; SD = 1.4; Control group: M = 15.4; SD = 1.2	33 in NSSI group +88 in control group	NSSI	Yes	based on clinical data	Scores on the questionnaires used (measuring internalizing, externalizing and other psychological problems) were significantly higher among those who engaged in NSSI than in the control group. Habitual self-injurers were more impulsive and alexithymic than occasional self-injurers, but they sought for help more frequently.	Cases had significantly higher scores on externalizing problems in Child Behaviour Checklist (CBCL; Z = 6.42; *p* < 0.05) and in Youth Self-Report (YSR; Z = 4.57; *p* < 0.05), than controls.
Guendelman et al. (2016) [28]	USA	prospective, girls with ADHD, W1, W2 - after 5 years, W3 - after 10 years	W1: M = 9.6, SD = 1.7; W2: M = 14.3; W3: 19.710-year follow-up	140 girls with ADHD +88 in comparison group	NSSI	Yes	interview for NSSI; SIQ	Maltreated participants among ADHD diagnosed girls and comparsion group were significantly more impaired with respect to suicidal attempts, internalizing symptomatology eating disorder symptomatology, and well-being than nonmaltreated participants.	There were no signifficant differences in engaging in NSSI between maltreated and non-maltreated participants among ADHD diagnosed girls and comparsion group. Odds ratio: 1.19 (CI 95%: 0.78–1.80); non significant
Guertin et al. (2001) [29]	USA	cross-sectional, clinical sample, inpatients after suicide attempt	12–18; M = 15.1; SD = 1.5	95	self-mutilation	Yes	questionnaire for self-mutilation; FASM	In the self-mutilative (and suicide attempt too) group, diagnoses of ODD, MDD, dysthymia were more common, they also had higher scores on hopelessness, loneliness, anger, risk taking and reckless behaviour and alcohol use scales.	ODD was significantly more common in the self-mutilative + suicide attempt group, than in the suicide attempt only group (X^2^ = 3.91; *p* < 0.05).
Hinshaw et al. (2012) [30]	USA	prospective, girls with ADHD, matched controls, BL, % years FU, 10 years FU	BL: 6–12; 10 years FU: 17–24; M = 19.6	BL: 221; 10 years FU: 216	self-injury	Yes	questionnaire for self-injury; SIQ	Participants with childhood ADHD had higher rates of ADHD and comorbidity, more serious impairment, suicide attempts and self-injury. There were no differences in eating pathology, substance use and driving behaviour. There were significant differences between inattentive and combined type only in suicide attempts and self-injury.	Self-injury is significantly more common among girls diagnosed with combined ADHD in childhood. Odds ratio: 4.5; *p* < 0.05.
Hurtig et al. (2012) [31]	Finland	birth cohort (9479), subsample of that	16–18	104 + 169	DSH	Yes	part of the interviewing process questions for DSH	ADHD group had both more suicidal ideation and more DSH than the non-ADHD group. Other factors in suicidal behaviour: female gender, childhood emotional and behavioural problems, concurrent depression and anxiety, and especially in DSH behavioural disorder, substance use disorder and strain in family relations.	ADHD prevalence was significantly higher in DSH group than in no-DSH group (69% vs. 32%, *P* < 0.001).
Ilomaki et al. (2007) [32]	Finland	cross-sectional, CD population: alcohol dependents and non-dependents	12–17; alcohol dependent girls: M = 16.4; boys: M = 16.3; non-dependent girls M = 15.3; boys: M = 15.1	141	self-mutilation	Yes	part of the interviewing process questions for DSH	40.7% of CD girls and 29.3% of CD boys suffered from alcohol-dependence as well. Life-threatening suicide risk is higher among dependent girls and boys. Self-mutilation risk is also elevated in the group of dependent girls and in the group of dependent boys as well.	Prevalence of repeated self-mutilation is high among CD patients and higher among girls. The prevalence rate depends on gender and alcohol dependence of participants, the lowest was among non-alcohol-dependent boys, and the highest was among alcohol-dependent girls. Odds ratio among girls: 3.9 (CI 95%: 1.1–13.8); Odds ratio among boys: 5.3 (CI 95%: 1.1–26.5); *p* < 0.05.
Izutsu et al. (2006) [33]	Japan	cross-sectional, normal population	boys: M = 14.2; SD = 0.7; girls: M = 14.2; SD = 0.7	486	DSH	Yes	own questionnaire for DSH	Lifetime prevalence of self-cutting: 8.0% among boys; 9.3% among girls; lifetime prevalence of self-hitting: 27.7% among boys; 12.2% among girls. Lifetime prevalence of tobacco use: 33.1% among boys, 14.3% among girls; lifetime prevalence of alcohol use: 74.1% among boys; 63.4% among girls. ADHD scores are significantly higher in all groups with these problems (self-cutting, self-hitting, tobacco use, alcohol use) than those who do not have problems in these areas.	ADHD scores are significantly higher in all groups with these problems (self-cutting, self-hitting, tobacco use, alcohol use) compared to those who do not have problems in these areas (*t* = 2.55–5.56; *p* < 0.05).
Jacobson et al. (2008) [34]	USA	retrospective study, clinical sample, outpatients	12–19; M = 15.1; SD = 1.7	227	DSH, NSSI, SA	Yes	interview for DSH; LPC	The most frequent method of DSH was cutting, followed by overdose, burning, and strangling self. 52% did not engage in self-injury, 13% engaged in NSSI, 16% engaged in SA, 17% engaged in both SA and NSSI. Major depressive disorder and/or posttraumatic stress disorder were more common in SA group, than in NSSI group. Borderline personality features were more frequent among those who engaged in any kind of DSH. The suicidal ideation levels of those in the NSSI group were similar to those in the No DSH group.	There were non-significant differences between the DSH group and the non-DSH group regarding disruptive behaviour disorders (X^2^ = 2.64; *p* = 0.45), and significant differences in borderline-features (X^2^ = 28.69; *p* < 0,001)
Jenkins et al. (2015) [35]	USA	cross-sectional, specific psychopatology group, IED patients, perosnality disorder, HC	18–81; M = 35.1; SD = 10.3	1097	NSSI	Yes	questionnaires for NSSI; DSHI	Individuals with personality disorders, and particularly with comorbid IED, are at increased risk for self-injurious behaviours. Traits of aggression, impulsivity and affect lability has an effect on the relationship between personality disorders, IED and self-injurious behaviours, especially NSSI.	Individuals with personality disorders, particularly with comorbid IED are at increased risk for self-injurious behaviours (IED - control: X^2^ = 9.89; personality disorder - control: X^2^ = 40.85; IED and personality disorder vs. control: X^2^ = 94.03; *p* < 0.05). Traits of aggression, impulsivity and affect lability has an effect on the relationship between personality disorders, IED and self-injurious behaviours, especially NSSI.
Keenan et al. (2014) [36]	USA	prospective normal population	13–14 years old girls	2180	NSSI	Yes	part of the interviewing process questions for NSSI	6.0% of the sample engaged in NSSI according to either child or parent report. In the ‘aggression’ domain, initial levels of conduct problems and self-control were significantly associated with NSSI. The initial levels of relational aggression were not associated with NSSI, but the increases in these levels over time were. In the ‘depression’ domain, initial levels of depression and a decrease in assertiveness were associated with later risk for NSSI. In the ‘environmental stressor’ domain, NSSI was associated with initial levels of peer victimization and negative life events. The most common pathway to NSSI was aggression combined with depression and environmental stressor domains, and the least common was environmental stressors only.	Higher initial levels of conduct problems (odds ratio: 1.08 (CI 95%: 1.01–1.15); *p* < 0.05), lower initial levels of self-control (0.92 (CI 95%: 0.87–0.98); *p* < 0.05) and increases in relational aggression over time (1.36 (CI 95%: 1.10–1.70); *p* < 0.05) were associated with NSSI.
Kirkcaldy et al. (2006) [37]	Germany	cross-sectional, clinical sample, inpatients	3–24; M = 13.4; SD = 3.4	3649	SIB	Yes	based on clinical data, regularly used: questionnaire for SIB, German Inventory	SIB and suicidal behaviours correlated, but aggression (or disruptive behaviour) was not correlated with suicide or SIB. Age and family disharmony were risk factors for suicide, but there was no association with disruptive behaviour and SIB. Intelligence and age were significant predictors of aggression among females.	There was no correlation between SIB and disruptive behaviour.
Lam et al. (2005) [14]	Australia	cross-sectional, patients owing injuries	Range: 5–15	18.729	self-harm	No	based on clinical data	Participants from the age group 11–15 years-old were more likely to be involved in intra- and interpersonal violence than those from the age group 5–10-years-old. Female gender showed a stronger association with suicide or self-harm, while socioeconomically advantaged background was related to interpersonal violence. Suicide attempts and engagement in self-inflicted injuries were associated with staying in hospital for longer time. 3 cases resulted in death.	Compared to other causes of injury, patients whose cause of hospital admission was suicide were more likely to be diagnosed with ADD, while patients with self-harm were also more likely to have comorbid ADD as compared to those with other types of injuries (odds ratio in univariate model: 3.76 (CI 95%: 1.73–8.15); odds ratio in multivariate model: 6.27 (CI 95%: 2.76–14.26)).
McCloskey et al. (2008) [38]	USA	cross-sectional, adults with intermittent explosive disorder	M = 36.10; SD = 9.32	376	SIB	Yes	own interview for SIB	16% reported self-aggression, 12.5% suicide attempts, 7.4% non-lethal self-injurious behaviours. MDD, drug dependence and Axis-I comorbidities all predicted SA or SIB in IED. Women were at increased risk for self-aggressive behaviour overall, but it was not significant for either SA or SIB.	16% of IED patients reported self-aggressive behaviour, with 12.5% attempting suicide and 7.4% engaging in SIB. This shows a relationship between IED and self-aggression.
Meza et al. (2016) [39]	USA	prospective, patients (girls) with ADHD and control group	W1, M = 9.6, range6–12); 5 years follow-up (W2, M = 14.2, range 11–18);10-year follow-up (W3, M = 19.6, range 17–24)	228 with (*n* = 140) and without (*n* = 88) childhood ADHD	NSSI	Yes	interview for NSSI; SIQ	Wave 1 commission errors (indicator of response inhibition) predicted Wave 3 suicide ideation with marginal significance and significantly predicted suicide attempts and NSSI severity. Social preference was a significant partial mediator of the Wave 1 commission errors and suicide ideation: indirect effect. Wave 2 peer victimization was a significant partial mediator in the Wave 1 commissions and Wave 3 NSSI severity.	There were a significant differences in NSSI severity between ADHD group and comparsion group (Cohen’s d:0.60; *p* < 0.001). Childhood response inhibition predicted NSSI in young adulthood in longitudinal study (B = 0,16; *p* < 0,05; R2 = 0.03).
Nock et al. (2006) [40]	USA	cross-sectional, case series, inpatient unit patients who engaged in NSSI	12–17 (M = 14,7)	89	NSSI, SIB	Yes	questionnaire for self-mutilation; FASM	87.6% met at least one Axis I criterion, internalization 51.7%, externalization 62.9%, substance use 59.6%; 67.3% of female patients met Axis II criteria (51.7% BPD was the most common). 70% reported at least one suicide attempt, 55% reported more than one suicide attempt.	Some sort of externalizing disorder was present in 62.9% of the NSSI sample (CD: 49.4%; ODD: 44.9%). Substance use disorder was present in 59.6%, while some sort of internalizing disorder was present in 51.7%.
Preyde et al. (2012) [41]	Canada	prospective, clinical population	5–18; M = 11.57 (SD = 2.75).	210 (Self-harm data available for 169)	NSSI, self-harm	Yes	part of the interviewing process questions for self-harm; BCFPI	39% who reported self-harm at admission were less than 12 years of age. Most of the differences between self-harmers and non-self-harmers on symptom severity at intake disappeared by the time of follow up. Youth who engaged in self-harm had higher symptom severity on Attention and Impulsivity regulation, Managing Mood, Internalizing Behaviour and Total Mental Health. At discharge, symptom severity only differed on the Total Mental Health subscale, and no differences were evident at 12 to 18 months post-discharge or 36 to 40 months post-discharge.	Higher symptom severity among self-harmers compared to non-self-harmers on Attention and Impulsivity regulation and Total Mental Health was measured. Mean scores (SD; CI 95%) on Attention and Impulsivity regulation: no self-harm: 70.9 (10.2; 68.8–73.0); self harm: 75.3 (7.8; 73.0–77.5); mean scores (SD; CI 95%) on Total Mental Health: no self-harm: 77.4 (11.4; 75.1–79.8); self harm: 82.5 (11.1; 79.2–85.9); *p* < 0.05.
Semiz et al. (2008) [42]	Turkey	cross-sectional, male offenders in psychiatry department of a military	20–36; M = 22.7; SD = 2.9	105	SIB	Yes	own interview for SIB	92% reported SIB with 57% reporting more than 10 episodes. The onset of SIB was between 5 and 23 years with mean onset at 14.8 years, and the mean duration of the behaviour was 7.2 years. 65% had received medical treatment for their SIB. APD participants who had comorbid ADHD did report a significant increase in suicide attempts but not in SIB or criminal behaviours. The number of ADHD symptoms was significantly correlated with frequency of SIB. ADHD total score was significantly correlated with frequency of SIB and negatively correlated with age at onset of SIB.	The number of ADHD symptoms was significantly correlated with frequency of SIB (correlation coefficient: 0.32; *p* < 0.05). ADHD total score was significantly correlated with frequency of SIB (correlation coefficient: 0.38; *p* < 0.05) and negatively correlated with age of onset of SIB (correlation coefficient: −0.23; *p* < 0.05).
Swanson et al. (2014) [43]	USA	prospective, patients (girls) with ADHD and control group	6–12; M = 9.1	140 girls with ADHD +88 in comparison group	NSSI	Yes	interview for NSSI; SIQ	Combined type of ADHD engaged in the most severe forms of NSSI using the most methods compared to childhood-diagnosed inattentive type of ADHD and comparison groups. Both ADHD subtypes showed increased NSSI frequency compared to the comparison group but did not significantly differ from each other. Persistent ADHD showed increased NSSI compared to those with transient ADHD or the comparison group. Participants with persistent ADHD also had a higher rate of suicide attempts. Externalizing symptoms and a lab-based measure of response inhibition/impulsivity were significant partial mediators between ADHD and NSSI while internalizing symptoms during adolescence was a significant partial mediator of the ADHD-suicide attempt linkage.	There were significant differences in NSSI frequency, variety and severity between combined ADHD and comparsion groups (inattentive ADHD and control; Cohen’s d: 0.20–0.85; *p* < 0.05). Persistent ADHD also differed significantly from comparsion groups (transient ADHD and control) in NSSI frequency, variety and severity (Cohen’s d: 0.32–0.87; *p* < 0.05). Externalizing symptoms mediate between ADHD and NSSI (IE = 0.29; SE = 0.11; CI 95%: 0.10–0.51).
Taylor et al. (2014) [44]	New-Zealand	case–control study, patients with retrospective ADHD and control group	M = 31.9; SD = 1.6	66 (35 with adult ADHD and 31 with no ADHD)	DSH	Yes	questionnaire for DSH; DSHI	Significant associations were present between ADHD symptom severity and history of self-harm behaviour, suicidal ideation and suicide attempts. These relationships were mediated by comorbidity (mood, anxiety, drug and alcohol abuse disorders) and emotion-focused coping style. The results suggest that comorbid mental health disorders and emotion-focused coping might mediate the relationship between self-injurious behaviour and ADHD.	Significant associations were present between ADHD symptom severity and history of self-harm behaviour (B = 0.52; SE = 0.24; *p* < 0.05). These relationships were mediated by comorbidity (B = 0.68; SE = 0.23; *p* < 0.01) and emotion-focused coping style (B = 0.72; SE = 0.22; *p* < 0.01).
Vaughn et al. (2015) [15]	USA	prospective, normal population	18–49	19.073	DSH	No	part of the interviewing process questions for self-harm	DSH was linked to ethnicity since African-American, Latinos and Asians were less likely to report DSH than Whites. DSH was associated with all forms of child maltreatment: child sexual abuse, physical abuse, child neglect and exposure to serious conflict in the home. DSH was also associated with lifetime victimization: intimate partner violence, violent victimization, and to ever having been stalked. DSH was also associated with MDD, avoidant personality disorder and schizotypal personality disorder. DSH was also related to engaging in violent behaviours, particularly robust effects were shown with “having forced someone to have sex with you against their will”.	Besides DSH being linked to substance abuse (odds ratios: 2.90–7.02), it was also associated with violent behaviours such as robbery, intimate partner violence, forced sex, cruelty to animals and use of a weapon (odds ratios: 3.32–12.73).
You et al. (2011) [45]	Hong Kong	cross-sectional, normal population	11–19; M = 14.7; SD = 1.9	6374	NSSI	Yes	part of a longer questionnaire, questions for NSSI	Repetitive self-injurers had more impulse-control and emotional problems, than episodic self-injurers. Severe self-injurers were more impulsive than mild self-injurers. The frequency and severity of NSSI are two possible dimensions that distinguish NSSI subgroups.	There was a strong link between NSSI frequency (odds ratio: 1.37 (CI 95%: 1.32–1.42); *p* < 0.01) and severity (1.12 (CI 95%: 1.08–1.17); *p* < 0.01) and impulse control problems.
Young et al. (2009) [16]	UK	cross-sectional, prisoner population	M = 30.0; SD = 8.2	198	critical incident, self-injury	No	based on institutional data	24% of prisoners met ADHD criteria in the present or in the past. 23% of them were fully symptomatic, 33% were in partial remission and 44% were in full remission. Critical incidents (verbal and physical aggression, damage to property, self-injury, and severity of aggression) were more common among the ADHD group than in the non-ADHD group, the most severe forms found among fully symptomatic ADHD group.	The mean number of critical incidents was significantly higher in the ADHD group than in the non-ADHD group (F = 5.01; *p* < 0.001).
Zlotnick et al. (1999) [17]	USA	cross-sectional, psychiatric outpatients	M = 40.6; SD = 14.0	256	self-mutilation	No	own questionnaire for self-mutilation	From the Axis-I disorders, substance abuse, posttraumatic stress disorder and IED showed significant association to self-mutilative behaviour, independent of BPD and antisocial personality disorder. Moreover, higher scores of dissociation were associated with self-mutilation while controlling for age, BPD, sex, education, sexual abuse and physical abuse.	Self-mutilation was associated with substance abuse (X^2^ = 13.73; *p* < 0.05), IED (X^2^ = 6.35; *p* < 0.05) and antisocial personality disorder (X^2^ = 8.03; *p* < 0.05).

The methodology of this systematic descriptive review follows the PRISMA guidelines.

## Results

*Summary of included papers in the review. Abbreviations: ADD: attention deficit disorder; ADHD: attention deficit hyperactivity disorder; BL: baseline; BPD: borderline personality disorder; CD: conduct disorder; DSH: deliberate self-harm; DSHI: Deliberate Self Harm Inventory; DSP: deliberate self-poisoning; EDR: electrodermal responding; FASM: Functional Assessment of Self-Mutilation; FU: follow-up; IED: intermittent explosive disorder; LPC: Lifetime Parasuicide Count; L-SASI: Lifetime-Suicide Attempt Self-Injury; MDD: major depressive disorder; NSSI: nonsuicidal self-injury; ODD: oppositional defiant disorder; PEP: pre-ejection period; RSA: respiratory sinus arrhythmia; SA: suicide attempt; SIB: self injurious behviour; SII: self inflicted injury; SIQ: The suicidal ideation questionnaire; SITBI: Self-Injurious Thoughts and Behaviors Interview.*

### Terms and definitions for self-injurious behaviours in the included studies

Below, the different terms and their presence in the reviewed publications are displayed. It is important to clarify that in general the term ‘self-injurious behaviours’ is used for describing all of these acts, which are not the same as the specific term ‘self-injurious behaviour (SIB)’.

In 7 papers there was no clear distinction between the purpose of the investigated self-injurious act (whether it was with suicidal intent or not) [11–17], but in the remaining 28 papers the defined self-injurious behaviour was a nonsuicidal act [18–45].

There were 11 different terms for self-injurious behaviour in the investigated 35 papers.

‘NSSI’ was the most common term: this expression was presented in 10 studies [27, 28, 34–36, 39–41, 43, 45]. In accordance with the term itself this expression was used only in nonsuicidal meaning. Six studies used only this term for describing the behaviour [27, 35, 36, 39, 43, 45]. In other studies, NSSI could be found as a part of wider self-injurious behaviours: Jacobson et al. [34] use ‘deliberate self-harm’ (DSH), Nock et al. [40] use ‘self-injurious behaviour’ (SIB), Preyde et al. [41] and Guendelman et al. [28] use ‘self-harm’ for describing both suicidal and nonsuicidal self-injurious behaviours. Darke and Torok [22] used ‘non-suicidal self-harm’ as a term, but their definition was similar to other NSSI definitions.

‘DSH’ was presented in 6 publications [15, 20, 31, 33, 34, 44]. Four studies used it in a nonsuicidal meaning [20, 31, 33, 44], however, 2 studies [15, 34] did not make this distinction. Five papers [15, 20, 31, 33, 44] defined ‘DSH’ as physical self-injurious behaviour, although Jacobson et al. [34] defined it as a possible overdose act as well. Both Aglan et al. [11] and Chou et al. [12] used the term ‘deliberate self-poisoning’ (DSP), which they originated from DSH, but only in the meaning of an overdose, they did not make the suicidal-nonsuicidal distinction.

‘Self-mutilation’ was found in 5 publications [17, 19, 25, 29, 32]. The term is used for nonsuicidal physical self-injurious behaviours, except for Zlotnick et al. [17], who did not distinguish the purpose of the act.

‘SIB’ was a term in 4 papers [37, 38, 40, 42], it is also mainly used in a nonsuicidal meaning, except for Nock et al. [40].

‘Self-injury’ was presented in 4 papers [18, 24, 30, 45], and ‘self-harm’ was found in 3 studies [14, 23, 41]. Young et al. [16] used ‘self-injury’ as a subgroup of ‘critical-incidents’ that they defined as auto or hetero-aggressive acts in an incarcerated population.

There were some other phrases describing self-injurious behaviour: ‘parasuicide’ [13]: there is no distinction between suicidal-nonsuicidal intent; ‘self-inflicted injury’ (SII) [21]: this is a nonsuicidal act; ‘suicide gesture’ [26]: this is a nonsuicidal self-injury or a suicidal attempt to communicate with others.

### Measurements of self-injurious behaviours in the included studies

Among the studies included in our review, diagnostic interviews, self-reported questionnaires and institutional records were used to measure self-injurious behaviours: altogether we found 20 different instruments among the investigated 35 papers.

In 9 cases, the authors designed a longer diagnostic interview, and some questions about self-injurious behaviours were included in it [11, 15, 18, 19, 22, 31, 32, 36, 41].

An interview specially developed for measuring self-injurious behaviours was used in 10 studies [13, 21, 26, 28, 30, 34, 38, 39, 42, 43]. The Self-Injurious Thoughts and Behaviors Interview (SITBI) [46] was applied by Garcia-Nieto et al. [26]. The Lifetime Parasuicide Count (LPC) [47] was used by Jacobson et al. [34], Crowell et al. [13], Crowell et al. [21]. However, in the study of Crowell et al. [21], it was mentioned under a different name: Lifetime-Suicide Attempt Self-Injury (L-SASI). An interviewer-administered modification of the Self-Injurious Questionnaire (SIQ) [48] appeared in the studies of Guendelman et al. [28], Hinshaw et al. [30], Meza et al. [39] and Swanson et al. [43], however these 4 papers are based on the same population. Both McCloskey et al. [38] and Semiz et al. [42] developed their own interview as an instrument for measuring self-injurious behaviours.

Self-report questionnaires were used for measuring self-injurious behaviours in 9 studies [17, 20, 23, 29, 33, 35, 40, 44, 45] in all. Two papers contained question(s) about self-injurious behaviours as a part of a longer questionnaire [23, 45]. Individual questionnaires as an instrument for the measurement of self-injurious behaviours can be found in 7 publications [17, 20, 29, 33, 35, 40, 44]. The Deliberate Self-Harm Inventory (DSHI) [49] appears in 3 studies [20, 35, 44]. The Functional Assessment of Self-Mutilation (FASM) [50] was used in 2 studies [29, 40]; the original questionnaire form of SIQ [51] was not applied by any of the investigated papers. Own developed questionnaire was used in 2 papers [17, 33].

In the remaining 7 publications, some kind of institutional (health care, prison) records or national databases were the sources of the data on self-injurious behaviours [12, 14, 16, 24, 25, 27, 37]. It is important to highlight the work of Kirkcaldy et al. [37]: although they based their examinations on the health records of German adolescents and youths, in the region where their study was conducted, the German Inventory Questionnaire – which contains questions about SIB – is a regular part of the documentation.

### Prevalence of self-injurious behaviour

Epidemiological data about self-injurious behaviours in different groups can be found in 20 publications (see details below).

Six studies examined the normal population [15, 20, 23, 33, 36, 45]. Among them 3 papers used the term DSH and found the prevalence rates between 2.9 and 41.9% [15, 20, 33]. NSSI was a term in 2 publications: with prevalence rates of 6% [36] and 15% [45]. Evren et al. [23] used the term ‘self-harm’, and found its prevalence to be 14.3%.

Five studies investigated general psychiatric populations (inpatients, outpatients or intensive home-based treatment population) [17, 24, 34, 37, 41]. Among them only one paper used NSSI as a term: Jacobson et al. [34], however they also used DSH to include suicidal behaviour, and they found 13% prevalence of NSSI and 17% of DSH. Feingold et al. [24] investigated ‘self-injury’, and found its prevalence to be 32.4%. Kirkcaldy et al. [37] examined SIB with a prevalence rate of 59%. Preyde et al. [41] studied ‘self-harm’ and found 34% as its prevalence rate. ‘Self-mutilation’ was the term in the study of Zlotnick et al. [17], and the prevalence rate was 33.2%.

In the study of Carli et al. [19] there was an incarcerated population. They found the prevalence of ‘self-mutilation’ to be 17.0%.

In the remaining 8 studies, the aim of the investigation was to compare a group having a specific psychopathology with a healthy control group or with a group having another psychopathology.

Four publications examined attention deficit hyperactivity disorder (ADHD) as a group with specific psychopathology [12, 18, 30, 31]. Among ADHD patients versus non-ADHD patients, there was a robust difference in the prevalence rates of self-injurious behaviours. Prevalence of self-injury was 50.6% in the combined type of ADHD, versus 28.9% in the inattentive type of ADHD, versus 19% in the non-ADHD control group [30]. Prevalence of self-injury among drug dependent patients with ADHD was 47.7%, versus 25.2% without ADHD [18]. Prevalence of DSH was 69% in an ADHD group and 32% in a control group [31]. In one study there was a significant difference in the incidence of DSP: it was 6.13/10,000 persons/years in the ADHD group and 1.36/10,000 persons/years in the control group [12].

Two studies examined intermittent explosive disorder (IED) as a specific psychopathology. McCloskey et al. [38] found the prevalence of SIB to be 16% in this group. Jenkins et al. [35] investigated participants with personality disorder with or without IED: they found the prevalence of NSSI to be 18%.

Only in one publication could data be found on the prevalence of self-mutilation among participants with conduct disorder (CD) [32]: they found its prevalence between 15.5 and 62.5%. It depends on the gender and alcohol dependence of participants, with the lowest prevalence being among non-alcohol-dependent boys, and the highest being among alcohol-dependent girls. However there was another study, which examined the prevalence of SIB among psychiatric offenders in a military institution with antisocial personality disorder [42]: the prevalence rate of SIB was 92.0%.

### Correlations of self-injurious behaviours and externalizing psychopathology

Fifteen publications investigated the associations between self-injurious behaviours and ADHD [12, 14, 16, 18, 21, 23, 25, 28, 30, 31, 33, 41–44]. Four of them studied ADHD versus non-ADHD groups, and found robust differences in the prevalence or incidence rates of self-injurious behaviours (self-injury [18, 30]; DSH [31]; DSP [12]). Higher symptom severity or higher scores on ADHD symptom scales or higher numbers of ADHD symptoms are associated with self-injurious behaviour in 6 papers: ADHD scores were higher in the SII group than in the control group or depressed group [21]; ADHD scores were higher in the self-harm group versus non-self-harm group [23]; and in the DSH group versus non-DSH group [33]; frequency of SIB was correlated with the number of ADHD symptoms [42]; those who engaged in self-harm in a residential treatment or intensive home-based treatment programme had higher ADHD symptom severity than those who did not engage in this behaviour [41]; and there was a significant association between symptom severity of ADHD and history of DSH behaviours [44]. Moreover, Fulwiler et al. [25] found the importance of childhood ADHD in adulthood self-mutilation among prisoners. Lam et al. [14] underline that the odds ratio of having comorbid attention deficit disorder (ADD) is 3 times higher in the self-harm group than in the non-self-harm group. Swanson et al. [43] found that both the inattentive and combined type of ADHD were associated with NSSI, however more severe forms and higher frequency of NSSI are more likely to be associated with the combined type. Young et al. [16] emphasize that the number of critical incidents (including self-injury) were higher among people with ADHD in an incarcerated population. According to the work of Guendelman et al. [28], maltreatment is a strong risk factor of both NSSI and any other self-harm acts (suicide attempts), and there is no significant difference in this risk effect between girls diagnosed with ADHD and comparison group.

Twelve publications examined the possible connection between conduct disorder (CD) or CD symptoms or antisocial personality disorder in adulthood and self-injurious behaviours [11, 15, 20–22, 26, 32, 34, 36, 37, 40, 42]. Seven of them found significant positive associations: CD is a risk factor of adulthood DSP repetition [11]; both CD and oppositional defiant disorder (ODD) correlated with DSH, however there is no significant difference in the level of correlation between them [20]; there were higher CD scores in the SII group than in the control group or depressed group [21]; antisocial personality disorder is a risk factor of suicidal gestures (included self-injurious behaviour) [26]; prevalence of self-mutilation is high among CD patients, especially when they are girls and they are also alcohol-dependent [32]; higher initial levels of conduct problems are associated with NSSI [36]; and DSH is linked to violent behaviours (robbery, intimate partner violence, forced sex, cruelty to animals, use of a weapon) [15]. Two publications described a non-significant positive connection: CD symptoms are more frequent in a non-suicidal self-harm group than in the control group [22]; frequency of any disruptive behaviours was higher both in DSH and in NSSI groups than in the control group [34]. Nock et al. [40] found the presence of CD to be 49.4% in their 100% NSSI sample. Semiz et al. [42] found the prevalence of SIB to be 92% in a 100% antisocial personality disorder sample. However Kirkcaldy et al. [37] could not find any association between SIB and disruptive behaviour.

Four papers studied the link between oppositional defiant disorder (ODD) and self-injurious behaviours or SIB. Cerutti et al. [20] found, that ODD correlated with DSH but they did not find a significant difference with the correlation of CD and DSH. In the study of Crowell et al. [21], there are higher ODD scores in the SII group than in the depressed group or control group. Guertin et al. [29] found that ODD was more common in both the self-mutilator and suicide attempter group than in the suicide attempter only group. In Nock et al. [40], in the 100% NSSI sample the presence of ODD was 44.9%.

There are 3 publications that strengthen the relationship between intermittent explosive disorder and self-injurious behaviours (NSSI [35]; SIB [38]; self-mutilation [17]).

There was information about general externalizing symptoms in 5 publications. Crowell et al. [13] examined parasuicidal versus non-parasuicidal groups; in the parasuicidal group the T-scores of externalizing symptoms were significantly higher. Crowell et al. [21] investigated the SII group, control group and depressed group: T-scores of externalizing psychopathology were also significantly higher in the SII group than in the control or depressed groups. In the 100% NSSI sample of Nock et al. [40] the presence of any externalizing disorder was 62.9% while the presence of any internalizing disorder was 51.7%. Feingold et al. [24] did not study the direct link between externalizing pathology and self-injury, but they found a strong link between alcohol abuse and self-injury, and also described the association between externalizing pathology and alcohol abuse. Gatta et al. [27] examined neuropsychiatric inpatients who engaged in NSSI and controls from high schools. They found significantly higher externalizing scores in the NSSI group than in the control group according to both self-report and parent-report.

In the remaining 3 studies, the authors examined traits of externalizing psychopathology: Carli et al. [19] found a significant difference in the prevalence of self-mutilation between high-impulsive and low-impulsive groups. You et al. [45] investigated the role of impulse-control problems in the frequency and severity of NSSI and found individuals with more impulse-control problems had more severe and frequent NSSI behaviour. Meza et al. [39] found the importance of response inhibition in the prediction of NSSI among individuals with or without ADHD.

### Self-injury and suicide

In total 8 of the 35 articles assessed the relationship between self-injury and suicide [22, 25, 26, 29, 31, 32, 34, 43]. To compare results by age groups, we will first overview 3 papers that studied adult samples [22, 25, 26] and then turn to the other 4 studies that assessed adolescence populations [29, 31, 32, 34] and one longitudinal study that assessed young girls (M = 9.1 years) with 5 and 10 years follow-ups [43].

In their study on adult injecting drug users, Darke and Torok [22] assessed if the two phenomena (NSSI and suicidality) overlap. They found that 42.3% of those who reported NSSI also reported suicide attempt, and 39% of those who reported suicide attempt had also engaged in NSSI. In 83.3% of the cases with both suicidal and NSSI, the NSSI was present prior to suicide attempts.

The other two studies on adult samples focused on the differences between suicidal and NSSI behaviours [25, 26].

In an adult prisoner sample, Fulwiler et al. [25] found differences between those committing suicide attempts and self-mutilation regarding their methods (more lethal methods in suicide attempt) and frequency (higher frequency in case of self-mutilation). The two groups also differed in psychiatric comorbidities: diagnosis of an affective disorder was present in 87% of the suicide attempt group compared to 19% in self-mutilators, while a syndrome of mixed anxiety/dysthymia patterns present from childhood or adolescence (56% in self-mutilation group) and history of childhood hyperactivity (75% vs 7%) characterized the self-mutilation group.

Garcia-Nieto et al. [26] also focused on the differences between self-injurers with and without intent to die. Their results pointed out the differences in the prevalence of certain personality disorders: histrionic personality disorder (HPD) (0% in neither gestures nor attempts; 46.7% in gestures; 1.6% in attempt, 12.5% in attempts + gestures group) and antisocial personality disorder (APD) (5.3% in neither gestures nor attempts; 6.7% in gestures; 4.8% in attempt, 1.2% in attempts + gestures group) as risk factors were associated with suicide gestures. Narcissistic personality disorder (NPD) (27% in suicide attempt group, 0% in all other groups) showed association with suicide attempts, and BPD (7% in neither gestures nor attempts; 33.3% in gestures, 9.7% in attempts, 43.8% in gesture + attempt group) seemed to be a risk factor for both suicide gestures and attempts.

In an adolescent community sample [31], those with ADHD reported more suicidal ideation (57% vs. 28%, *P* < 0.001) and DSH (69% vs. 32%, *P* < 0.001) than those who did not have the disorder. Female gender and depression/anxiety were also associated with both suicidal ideation and DSH, while behavioural disorder, substance use and certain family factors (e.g. living in non-intact family, living in family with financial problems) were associated with DSH but not with suicidal ideation and acts.

In an adolescent inpatient sample, those who not only had a history of suicide attempt but also a history of self-mutilative behaviour were significantly more likely to have a diagnosis of ODD (χ2_1_(*N* = 75) = 3.91, *p* < .05); major depressive disorder (MDD; χ2_1_(*N* = 76) = 13.74, *p* < .01;) and dysthymia (χ2_1_ (*N* = 76) = 4.82, *p* < 0.05), and had higher scores of loneliness (F = 8.24, *p* < 0.05), anger (F = 11.88, *p* < 0.05), risk taking (F = 16.31, *p* < 0.05) and reckless behaviour (F = 7.21, *p* ≤ 0.005) [29].

Jacobson et al. [34] studied an adolescent outpatients sample with no DSH (52%), NSSI only (13%), suicide attempt only (16%) and with both NSSI and suicide attempt (17%). They found that the only psychiatric diagnosis in which those engaging in NSSI differed from those who had not engaged in any type of DSH was BPD. When distinguishing between DSH groups, differences were found in the likeliness of comorbid depression, posttraumatic stress disorder (PTSD) and in the levels of suicidal ideation. Regarding MDD, with the NSSI only group as the comparison group and controlling for gender, the suicide attempt only group (odds ratio – OR =3.43; 95% CI = 1.17–10.00; *p* = 0.03) and the suicide attempt and NSSI group (OR = 3.55; 95% confidence interval – CI = 1.24–10.16; *p* = 0.02) were more likely to have depression. Regarding PTSD, when controlling for gender, participants in the suicide attempt only group (OR = 8.93; 95% CI = 1.04–76.92; *p* = 0.046) and the suicide attempt and NSSI group (OR = 10.09; 95% CI = 1.18–85.97; *p* = 0.035) were more likely to have PTSD diagnosis than those in the NSSI-only group. The NSSI-only group also reported significantly lower levels of suicidal ideation scores than the other DSH groups: Suicide attempt only (adjusted mean difference = −20.24, *p* < 0.001) and suicide attempt and NSSI group (adjusted mean difference = −25.92; *p* < 0.001). Meanwhile the two other DSH groups (suicide attempt only and suicide attempt and NSSI groups) did not significantly differ from each other regarding the rates of MDD, PTSD and the levels of suicidal ideation.

Swanson et al. [43] studied girls recruited from both a clinical and non-clinical background in their follow-up study (age at baseline: M = 9.1 years; age at Wave 2: M = 14.2 years; age at Wave 3: M = 19.6 years). According to their findings, externalizing symptoms and inhibition/impulsivity might be significant partial mediators between Wave1 ADHD and W3 NSSI scores (indirect effect – IE = 0.29; standard error – SE = 0.11; CI95 = 0.10–0.51); while Wave 2 internalizing symptoms might be a significant partial mediator of the Wave 1 ADHD Wave 2 suicide attempt linkage (IE = 0.11; SE = 0.05; CI95 = 0.03–0.25).

Ilomaki et al. [32] did not investigate the association between the two phenomena but they found that alcohol dependence was a common potential risk factor for both phenomena since it increased the risk for both suicide attempts 3.8-fold [CI95 = 1.06–13.44; *p* = 0.041] and self-mutilation 3.9-fold (CI95 = 1.09–13.76; *p* = 0.037) among adolescent girls with DSM-IV-diagnosed CD.

## Discussion

In the current systematic review, 35 papers examining the correlation between externalizing psychopathology and self-injurious behaviours are summarized.

Consistent with the review on internalizing pathology and NSSI [7], we found wide-ranging terminology for self-injury: 11 different terms were used in 35 papers. Among these terms NSSI has the clearest definition, suggested by the ‘International Society for the Study of Self-injury’ (ISSS) in 2007 [2] and by DSM-5 [1]. DSH is the most unstable term, because it has many definitions, some authors use it only in a nonsuicidal meaning [20, 31, 33, 44], while others use it as non-fatal but with both suicidal and nonsuicidal meanings [15, 34]. The other issue is that self-poisoning was not included in the definition by most of the authors [15, 20, 31, 33, 44], but Jacobson et al. [34] use the term DSH both for physical self-damage and overdose cases as well. Moreover, DSP appeared as an individual term for self-poisoners [11, 12]. SIB is mostly defined as both suicidal and non-suicidal self-injurious behaviour, but self-poisoning is not included in the definition [37, 38, 40, 42]. Summarizing the main issues of defining self-injurious behaviour are the following: whether the definition used should include self-poisoning or not, and whether it should be clarified as a suicidal or a nonsuicidal act. For example, Kapur et al. [52] questioned the concept of NSSI, because in some cases it is impossible to decide if the act was with suicidal intent or not, especially in an adolescent population. Nock [53] drew attention to this definition problem in his earlier review; while sometimes those who have engaged in self-injury cannot themselves clarify their intent, conceptually it is important to make this distinction. Brunner, Kaess et al. [10] made the distinction between self-poisoning and self-harming by focusing on the surface of the body.

The heterogeneity that is present in the terminology and definitions of self-injurious behaviours is also reflected in the diversity of instruments used to assess the phenomenon. The most frequently used instruments (LPC [13, 21, 34]; DSHI [20, 35, 44]) were present in no more than three plus three out of the 35 studies. Among the studies included in our review, diagnostic interviews, self-reported questionnaires, institutional (health care, prison) records and national databases were used to measure self-injury. In more than half of the papers (18/35) the method was an interview, or special questions as part of a longer interview [11, 13, 15, 18, 19, 21, 22, 26, 28, 31, 32, 34, 36, 38, 39, 41–43]. The instruments were based on the previously described different definitions of self-injury and therefore they may measure somewhat different phenomena. This may be one of the factors that make it difficult to compare the results of different studies, as we have already highlighted above.

The prevalence rates of different self-injurious behaviours vary greatly both in clinical samples (13.0–59.0%) and in normal population based samples (2.9–41.9%). This variance can be explained partially by the definition and measurement heterogeneity, described above. However, using one term (e.g. NSSI) does not lead to more homogenous prevalence rates. In the specific psychopathology groups investigated, the diversity of prevalence rates could also be observed. Due to this heterogeneity, and with the aim of avoiding confusion, the original terms used by the authors of the papers are used in the ‘Results’ section, and the prevalence rates are mentioned in parallel. These various results underline the importance of standardized terminology and measurement for self-injurious behaviours, which would make the results of future studies comparable.

There were significantly higher prevalence rates in ADHD groups than in control groups [12, 18, 30, 31]. Moreover, the papers found higher ADHD symptom severity in self-injurer groups [21, 23, 33, 41, 42, 44]. The most severe and frequent forms of NSSI can be found in the combined types of ADHD versus the inattentive type and the control group [43], that strengthen the role of impulsivity in self-injurious behaviours, which is an important risk factor of self-injurious behaviours. However, according to Hamza et al. [54], itis very complicated to measure, especially with self-reported questionnaires. Moreover, it seems that different aspects of impulsivity have different roles on self-injury. Mood-based impulsivity influences the initiation of self-injury, cognitive facets of impulsivity are related to the maintenance of self-injury and behavioural impulsivity is associated with self-injury under conditions of negative affect [55].

In addition to the finding that ADHD and self-injurious behaviours are strongly associated, which is in agreement with a previous review on this topic [8], we found evidences for the association between self-injurious behaviours and other externalizing psychopathology. There are high prevalence rates of self-injurious behaviours among patients with CD (15.5–62.5%, it depends on the gender and alcohol dependence of participants [32]. Semiz et al. [42] found the prevalence rate of SIB to be 92.0% among male offenders with antisocial personality disorder. Moreover, there were higher CD scores in the self-injurer group, than in the control group [21]; and in 6 further studies there are strong links between CD or disruptive symptoms and self-injurious behaviours [11, 15, 20, 26, 32, 36]. Only one paper from the 12 investigated could not find a link between CD and self-injury [37]. The relationship between self-injurious behaviours and ODD is underlined by 3 papers [20, 21, 29], and there is a connection with intermittent explosive disorder as well according to 3 studies [17, 35, 38]. General externalizing symptoms are also more frequent in self-injurer groups [13, 21]. Nock et al. [40] found higher externalizing comorbidity than internalizing in self-injurer groups.

Although the aim of this review was not specifically to explore the relationship between NSSI and suicidality, 8 out of the 35 articles contained results regarding this topic. Although the two phenomena might overlap [22, 34] and might have shared clinical characteristics such as BPD [26], depression and anxiety [31] or alcohol dependence [32], most of these articles focused on factors distinguishing between suicide and NSSI. Those with and without suicidal intent might show differences in the frequency and methods of their self-injuring, comorbid symptoms and personality disorders [25, 26]. Some results show that while externalizing symptoms are associated with NSSI [25, 43], internalizing symptoms might play a bigger role in suicidality [25, 34, 43]; while others found higher numbers of both internalizing and externalizing symptoms among those who reported both suicidal behaviour and NSSI [29, 31].

### Limitations

The main limitation of our review is the heterogeneity of both the terminology and instruments, as we mentioned above, which makes it hard to compare the results of the included papers. In most of the cases, the distinction that the act was with or without suicidal intent was clear, however, among these 35 papers there were 7 papers, where this distinction was not clarified [11–17]. The fact that similar terms for self-injurious behaviour were also measured with multiple instruments in different studies also complicates the understanding of the topic.

A further limitation is that we may be missing some relevant papers on this topic. Firstly, because we included only articles that are written in English. Secondly, we have chosen the standard method and searched for publications in large indexed literature databases. This led to 14 papers on the topic of ADHD and self-injury, however, Allely [8] used a less conservative search method and checked ‘Google Scholar’ as well and found 15 articles on the topic of ADHD and NSSI, which is a sub-topic of our review topic. There were small differences in our keywords as well, that could also be an explanation for the differences in our findings. They used ‘self-poisoning’ as a search word as well. Finally there were 5 overlapping publications [25, 30, 31, 33, 42], but they found 9 publications that we did not (5 of them fit our inclusion criteria as well, but they had not appeared in our search) and we found 3 articles in their time window, that they had not [18, 21, 41]. The remaining 5 publications we found miss their time window [12, 14, 23, 43, 44]. In spite of all these differences in the papers, Allely [8] also concluded that ADHD and self-injury have a strong association, as we did, so these results support each other. Finally, it is important to underline that our aim was not to focus only on ADHD but on the whole spectrum of externalizing disorders.

Another methodological limitation could be that our work is a narrative review. A meta-analytic review, or a full meta-analysis would be more useful to compare the results of these very heterogenous papers. To handle this limitation and make the comparisons easier, sample sizes, design of the original studies and statistical results are presented in Table 1.

Another limitation could be that since our focus in this review was to investigate the association between NSSI and externalizing pathology, the role of externalizing problems in the relationship between NSSI and suicidality might be overrepresented, while other important factors might remain hidden. A review focusing directly on the NSSI-suicidality relationship, including two of the articles discussed in this paper [29, 34] was just recently published [56]. According to their conclusion, the two phenomena are correlated and share similar vulnerabilities and risk factors, moreover, NSSI itself is found to be a risk factor for suicidal behaviour.

## Conclusions

In conclusion, reviewing these 35 papers on self-injury and externalizing psychopathology underlines the confusion in terminology on self-injurious behaviours, which could lead to difficulty in comparing studies on the topic. Based on our findings, we suggest the use of the term NSSI in future studies, as NSSI has the most precise definition. Furthermore, it became an individual diagnostic category in DSM-5 [1].

Summarizing the results of these papers – although the connection between internalizing pathology and self-injurious behaviours are well-studied [7] – there is a strong association between self-injurious behaviours and externalizing pathology and individual externalizing disorders as well. Based on this knowledge, it seems to be important to investigate the presence of parallel internalizing and externalizing pathology and its connection to NSSI, because it may help to identify the most endangered populations.

## Abbreviations

ADHD: Attention deficit hyperactivity disorder
APD: Antisocial personality disorder
BPD: Borderline personality disorder
CD: Conduct disorder
CI: Confidential interval
DSH: Deliberate self-harm
DSHI: Deliberate Self-Harm Inventory
DSM: Diagnostic and Statistical Manual of Mental Disorders
DSP: Deliberate self-poisoning
FASM: Functional Assessment of Self-Mutilation
HPD: Histrionic personality disorder
IE: Indirect effect
IED: Intermittent explosive disorder
ISSS: International Society for the Study of Self-injury
LPC: Lifetime Parasuicide Count
L-SASI: Lifetime-Suicide Attempt Self-Injury
MDD: Major depressive disorder
NPD: Narcissistic personality disorder
NSSI: Nonsuicidal self-injury
ODD: Oppositional defiant disorder
OR: Odds ratio
PTSD: Posttraumatic stress disorder
SE: Standard error
SIB: Self injurious behviour
SII: Self inflicted injury
SIQ: Self-Injurious Questionnaire
SITBI: Self-Injurious Thoughts and Behaviors Interview
